# Comparative Characteristics of Porous Bioceramics for an Osteogenic Response *In Vitro* and *In Vivo*

**DOI:** 10.1371/journal.pone.0084272

**Published:** 2013-12-31

**Authors:** Hye-Rim Lee, Han-Jun Kim, Ji-Seung Ko, Yong-Suk Choi, Myun-Whan Ahn, Sukyoung Kim, Sun Hee Do

**Affiliations:** 1 Department of Clinical Pathology, College of Veterinary Medicine, Konkuk University, Seoul, Korea; 2 Department of Oral and Maxillofacial Radiology, School of Dentistry, Kyung Hee University, Seoul, Korea; 3 Department of Orthopedic Surgery, College of Medicine, Yeungnam University, Daegu, Korea; 4 School of Materials Science and Engineering, Yeungnam University, Gyeongsan, Korea; University of Akron, United States of America

## Abstract

Porous calcium phosphate ceramics are used in orthopedic and craniofacial applications to treat bone loss, or in dental applications to replace missing teeth. The implantation of these materials, however, does not induce stem cell differentiation, so suitable additional materials such as porous calcium phosphate discs are needed to influence physicochemical responses or structural changes. Rabbit adipose-derived stem cells (ADSC) and mouse osteoblastic cells (MC3T3-E1) were evaluated *in vitro* by the MTT assay, semi-quantitative RT-PCR, and immunoblotting using cells cultured in medium supplemented with extracts from bioceramics, including calcium metaphosphate (CMP), hydroxyapatite (HA) and collagen-grafted HA (HA-col). *In vivo* evaluation of the bone forming capacity of these bioceramics in rat models using femur defects and intramuscular implants for 12 weeks was performed. Histological analysis showed that newly formed stromal-rich tissues were observed in all the implanted regions and that the implants showed positive immunoreaction against type I collagen and alkaline phosphatase (ALP). The intramuscular implant region, in particular, showed strong positive immunoreactivity for both type I collagen and ALP, which was further confirmed by mRNA expression and immunoblotting results, indicating that each bioceramic material enhanced osteogenesis stimulation. These results support our hypothesis that smart bioceramics can induce osteoconduction and osteoinduction *in vivo*, although mature bone formation, including lacunae, osteocytes, and mineralization, was not prominent until 12 weeks after implantation.

## Introduction

The role of biomaterials in medicine is evolving from the use of biologically passive materials that are applied in a purely structural role to one in which the material properties are selected so that they may orchestrate the process of tissue regeneration. Bioceramics have been widely used as bone substitutes in dentistry, as well as in orthopedic and reconstructive surgery [1]–[3]. Bioactive ceramics such as hydroxyapatite (HA) and calcium metaphosphate (CMP) are known to elicit specific biological responses at the interface of the materials, resulting in the formation of a strong bond between tissues and the materials. Certain bioceramics can enhance osteoblast proliferation as well as osteoblast differentiation [4]–[7]. Of the various calcium phosphates, HA has received considerable attention because its mineral composition is similar to that of natural bone. The natural mineral phase of bone contains a hard, ceramic calcium phosphate mineral salt phase called HA [8]. Periosteum-derived cell culture is carried out upon these scaffolds to simulate the 3D structure of bone tissue [9]–[12]. Additionally, demineralized and acellular bovine xenograft matrices are predominantly composed of collagen, which improves the biocompatibility and biodegradability properties of the scaffold, as well as cellular adhesion, differentiation, and growth of the new bone. A scaffold made with a combination of HA and collagen could enhance the ability to carry progenitor cells to bone defects by taking advantage of the agglutinating capacity of collagen and the osteoconductive properties of HA. Bone is formed by a series of complex events that involve a sequential cascade of matrix protein (type I collagen and non-collagenous proteins) production and their subsequent controlled calcification [13]–[15]. Alkaline phosphatase (ALP) is a marker of early osteogenic development [16]–[19] and acts as an initiator and regulator of the calcification process. In this study, we examined the cell proliferation and expression of bone-related genes in adipose tissue-derived stem cells (ADSC) and osteoblast-like MC3T3-E1 cells grown on three types of bioceramics with different phase compositions of calcium phosphate, including CMP, HA, and HA- collagen (col). In addition, we investigated the osteogenic potency of porous bioceramics. The osteoconductive and osteoinductive capacity for each bioceramic was determined by *in vivo* evaluation of scaffolds implanted within the osteo-defect or intramuscularly, respectively.

## Materials and Methods

### Bioceramic specimen preparation

CMP discs were processed using calcium phosphate monobasic (Duksan, Korea). To remove impurities and volatile materials, samples were calcined at 720°C. After calcination, the powder was ball-milled with alumina balls for 24 h and then dried in an oven at 60°C. CMP powder (1.41 g) was pressed using a cylindrical mold with 2 tons of pressure for 30 s. The CMP discs were then sintered at 900°C with 5°C/min increments of temperature for 2 h in air. HA discs were prepared using calcium phosphate tribasic (Yakuri, Japan) as a precursor. First, HA powder (1.72 g) was pressed using a cylindrical mold with 2 tons of pressure for 30 s. The green HA discs were then sintered at 1200°C with 5°C/min increments of temperature for 2 h in air. To introduce amino groups to the trifluoroacetic anhydride (TFAA)-treated samples, a 3-aminopropyltriethoxysilane (APTES) solution was added to samples that were then heat-treated at 95°C. Type I collagen was dissolved in 1% acetic acid solution and mixed with 0.25% N-hydroxysuccinimide (NHS) to yield an active carboxyl group. APTES-modified HA samples were then immersed in a collagen solution for 5 h. The collagen-grafted HA samples were rinsed with deionized water (DW) and were then dried at room temperature.

### Cell cultures and ADSC isolation

Mouse osteoblastic cells (MC3T3-E1, ATCC No. CRL-2593, Manassas, USA) were maintained in α-minimum essential medium (α-MEM; Gibco, Grand Island, NY, USA) supplemented with 10% fetal bovine serum (Gibco) and 1% antibiotics (Gibco). Differentiation induction medium was prepared by adding 5 mM A-glycerol phosphate (Sigma-Aldrich, St. Louis, MO, USA) and 50 ng/mL vitamin C (Sigma-Aldrich) to the culture medium [20], [21].

Samples of rabbit intra-articular adipose tissue from one animal were minced and washed three times with phosphate-buffered saline (PBS; Gibco). Minced adipose tissue specimens were treated with 0.1% collagenase (Sigma-Aldrich) for 1 h at 37°C in a shaking incubator. Liberated cells were passed through a sterile 70-μm Nylon mesh (BD Biosciences, Bedford, MA, USA) to remove undigested fragments, and isolated ADSC were washed three times with Dulbecco's modified eagle medium (DMEM; Gibco) supplemented with 10% fetal bovine serum (Gibco) and 1% antibiotics (Gibco) [22]–[24]. Bone induction medium was prepared by adding 1 nM dexamethasone, 2 mM β-glycerol phosphate, and 50 µM vitamin C (Sigma-Aldrich) to the culture medium [25], [26].

### Cell proliferation

The effect of bioceramics on the proliferation of MC3T3-E1 cells and ADSC was determined by the MTT formazan (3-(4,5-dimethylthiazol-2-yl)-2,5-diphenyl-tetrazolium bromide) assay using a commercially available kit (Roche Diagnostic, Basel, Switzerland). Briefly, cells were plated in 96-well plates at a seeding density of 2×10^2^ cells/well and were cultured in the presence of a bioceramics release medium for 1, 5, or 6 d. MTT solution was added to the cells for 4 h to permit the formation of a water-insoluble formazan dye. After solubilization using dimethylsulfoxide, the released formazan dye was quantitated at 595 nm using SUNRISE (TECAN, Salzbrug, Austria).

### ICP-AES analysis of ion extracts

In order to measure Ca^2+^ and PO_4_^3−^ release from CMP, HA and HA-col, one disc of each bioceramic was added to 10 mL growth medium (α-MEM or DMEM) or each control medium separately. The medium was then collected after 6 days of soaking. The concentrations of Ca^2+^ and PO_4_^3−^ in the growth medium or the osteogenic control medium were then measured by inductively coupled plasma atomic emission spectroscopy (ICP-AES; Varian, CA, USA). The ion concentrations were expressed as ppm [27]–[29].

### RNA extraction and semi-quantitative reverse transcription-polymerase chain reaction (RT-PCR)

MC3T3-E1 cells were incubated for 1, 3, and 6 d while ADSC were incubated for 1, 6, 10, and 21 d in the bioceramic release medium or in a differentiation medium under conditions similar to those used for the control. Total RNA was extracted from the cultured cells using RNAiso Plus (Takara, Shiga, Japan) according to the manufacturer's instructions. One microgram of total RNA was converted to complementary DNA by a reverse transcription reaction prior to the use of a PCR Premix Kit (Bioneer, Daejeon, Korea) in an Eppendorf Mastercycler^®^ (Eppendorf, Hamburg, Germany). Serial changes of target gene expression were evaluated using a semi-quantitative RT-PCR technique with the glyceraldehyde 3-phosphate dehydrogenase (GAPDH) gene used as a control. The PCR primer sets used for MC3T3-E1 cells and ADSC are listed in Table 1.

**Table 1:** Primer sequences used in these experiments.

(MC3T3-E1 cell)			
Target gene	Source	Sequence (5′–3′)	Predicted length (bp)
GAPDH	XM001476707.2	F) ACC ACA GTC CAT GCC ATC AC R) TCC ACC ACC CTG TTG CTG TA	450
Type I collagen	NM007742.3	F) CCT GGT AAA GAT GGT GCC R) CAC CAG GTT CAC CTT TCG CAC C	222
Type II collagen	NM001113515.2	F) CAG GCC TCG CGG TGA GCC ATG AT R) GTT CTC CAT CTC TGC CAC G	273
Rankl	AB036798.1	F) CGC TCT GTT CCT GTA CTT TCG AGC G R) TCG TGC TCC CTC CTT TCA TCA GGT T	590
Runx2	NM001145920.1	F) GTA TGA GAG TAG GTG TCC CG R) ACA TCC CCA TCC ATC CAC TC	183
ALP	AB473959.1,	F) CGG GAC TGG TAC TCG GAT AA R) TGA GAT CCA GGC CAT CTA GC	209
Osteocalcin	L24429.1	F) CTT GGT GCA CAC CTA GCA GA R) TTC TGT TTC CTC CCT GCT GT	208
MMP3	BC006725.1	F) GCT TTG AAG GTC TGG GAG GAG GTG R) CAG CTA TCT TCC TGG GAA ATC CT	850
MMP13	NM008607.2	F) CTT GAT GCC ATT ACC AGT C R) GGT TGG GAA GTT CTG GCC A	130

(ADSC)			
Target gene	Source	Sequence (5′–3′)	Predicted length (bp)
GAPDH	NM001082253	F) TCA CCA TCT TCC AGG AGC GA R) CAC AAT GCC GAA GTG GTC GT	304
Type I collagen	XM 002713800.1	F) CGC GAT GGT CAG CCT GGA CA R) CCG GGA GGG CCA GCA GGA CC	310
Type IIcollagen	D83228.2	F) GAC CCC ATG CAG TAC ATG CG R) AGC CGC CAT TGA TGG TCT CC	370
Rankl	AY753407.1	F) GAC AGC ACG CGC TGC TTC TAC A R) GCC ACA TCG AGC CAC GAA CCT TC	221
Runx2	D83228.2	F) CAG GCA AGC CAC GCT CGT GA R) CTC GTG CGT GCG CAG GTC T	180
ALP	NM000478	F) AAA CCG AGA TAC AAG CAC T R) GCC CAT TGC CAT ACA GG	478
Osteocalcin	NM001082200.1	F) AAG AGA TCA TGA GGA GCC TG R) AGG AAA CAA GCA CTG TGC AT	420
MMP3	NM001082280.1	F) CTG GAG GTT TGA TGA GAA GA R) CAG TTC ATG CTC GAG ATT CC	386
MMP13	NM001082037.1	F) GCA GCA GTC TCC AGG CAC GG R) TCA GGG ACC CCG CAT CTC GG	328

### Immunoblot analysis

MC3T3-E1 cells and ADSC that were incubated in bioceramics release media, and adhesion cultures from the control medium cells were scraped using RIPA buffer containing protease inhibitor cocktail tablets (Roche, Mannheim, Germany). Subsequently, the supernatant was centrifuged at 12,000 rpm for 10 min at 4°C to obtain soluble protein. The protein concentration was then determined using the NanoVue spectrophotometer (GE healthcare, Freiburg, Germany). Proteins of interest were immunoprecipitated from 500 μg of total cell lysates, following previously described standard protocols [30], [31]. Cell lysates were mixed with 1 μg of primary antibody and then incubated for 2 h at 4°C. Twenty microliters of resuspended protein A/G PLUS-Agarose was incubated at 4°C on a rocker platform for 2 h. The pellets were washed 3 times, carefully aspirated, and the supernatant was added to 50 μl of 5X sample buffer mixed into the agarose pellet. Immunoprecipitated proteins were resolved by SDS-PAGE and were transferred to PVDF transfer membranes. After being blocked with 3% bovine serum albumin, the membranes were incubated with antibodies for immunoblot analysis: Runx2, ALP, type I collagen, and MMP3. After washing in TBS, the membranes were incubated with horseradish peroxidase (HRP)-conjugated secondary antibodies. Specific binding was detected using the Super Signal West Dura Extended Duration Substrate (PIERCE, Rockford, IL, USA), and the blots were exposed to medical X-ray film (Kodak, Tokyo, Japan). The images of protein bands were scanned, and the band intensities were quantified using Image J software (National Institutes of Health, Bethesda, MD, USA).

### Characterization of the bioceramics

MC3T3-E1 cells and ADSC were seeded on bioceramics (CMP, HA, and HA-col) placed in 6-well plates at a seeding density of 1×10^6^ cells/well and were cultured for 6 or 10 d. The surface morphology of the bioceramics was observed by scanning electron microscopy (SEM) (JSM-6380, JEOL, Tokyo, Japan) after sputter-coating with gold particles on a Cressington Scientific Instruments 108 Auto Sputter Coater (Cranberry Tep., PA, USA). The accelerating voltage for SEM images was 15 kV [28], [32].

### Morphometric analysis

Cells were seeded (1×10^6^ cells) on glass slides placed in culture dishes as described above. MC3T3-E1 cells were incubated for 6 d while ADSC were incubated for 10 d in bioceramic release media or in differentiation media under conditions similar to those used for the control. Samples were fixed using Bouin's fixative solution (BBC Biochemical, Mount Vernon, WA) and were stained with 0.1% Fastgreen (Sigma-Aldrich, St. Louis, MO, USA) for 30 min. Excess staining solution was removed by rinsing with glacial acetic acid (Merck KgaA, Germany) for 30 min. Cell layers were then stained using the Picrosirius stain for 90 min, with excess stain removed by rinsing with deionized water. Picrosirius stains collagen fibrils, and staining is enhanced by using polarized light due to the birefringence of collagen. Imaging shows the presence of type I collagen (red, orange, and yellow) in cell layers [27]. Matrix mineralization in the presence of osteoblasts was determined by staining with Alizarin Red S. For qualitative and quantitative evaluation of matrix calcification, slides were fixed in 4% paraformaldehyde and were stained with 2% Alizarin Red S for 5 min at room temperature. Slides were then washed with dehydration in acetone and in acetone-xylene (1:1) solution. For the MC3T3-E1 cell and ADSC cultures, slides were stained with Von Kossa reagent for the detection of minerals and then incubated in 1% silver nitrate for 20 min under UV light. The silver nitrate solution was removed by rinsing with distilled water (3 washes), and with 5% sodium thiosulfate for 5 min. Calcified extracellular matrix was stained brown-black.

### Animals and experimental design

Male 6-week-old Sprague-Dawley rats (n = 15) weighing an average of 200 g were purchased from Orient Bio (Seongnam, Korea). All experimental protocols were approved by the Institutional Animal Care and Use Committee of Konkuk University (KU12033). The rats were housed in a room at 22±2°C and a 12-h light-dark cycle was applied. Feed (PMI Nutrition International, St. Louis, MO, USA) and water were supplied *ad libitum*. The bone-forming capacity of each bioceramic was determined using femur defects and intramuscular implants in the rat model. Pre-operatively, rats were anesthetized with tiletamine zolazepam (Virbac Korea, Seoul, Korea) and xylazine HCl (Bayer Korea, Kyungkido, Korea). Rats were divided into five groups: Control (defect alone), Vetbond (3M, St. Paul, USA), CMP, HA, and HA-col. For evaluation of osteoconductive potency, a 2-mm diameter cortical bone defect was created in the diaphysis of the femur using a dental drill [33], followed by the implantation of each of the prepared bioceramics including CMP, HA, and HA-col in the lesion. Defects were carefully and sufficiently lavaged using 0.9% saline. Further, osteoinductive potency was evaluated using intramuscular implantation of bioceramics for exophytic bone formation. Rats were sacrificed at 12 weeks post-operation, and specimens were evaluated using immunohistochemistry and RNA/protein extraction from paraffin-embedded tissues.

### Soft X-ray analysis for cortical bone

Rats were sacrificed and their excised femurs were imaged using soft x-ray to examine cortical defects and the newly formed surrounding tissues. The difference in the opacity of the peripheral region of each implant was evaluated by image analysis.

### Histopathology and immunohistochemistry

The specimens were fixed in 10% neutralized buffered formalin and were processed using a standard method and embedded in paraffin. Sections of 4-µm thickness were stained with hematoxylin and eosin (H&E), while intramuscular tissue samples were also stained with Masson's Trichrome staining for collagen fibers [34]. For immunohistochemistry, sections were deparaffinized and incubated in 3% hydrogen peroxide for 30 min. The following pri mary antibodies were used: an anti-ALP monoclonal mouse antibody (Sigma-Aldrich) and an anti-type I collagen polyclonal goat antibody (Santa Cruz Biotechnology, CA, USA). The antigen-antibody complex was visualized using an avidin-biotin-peroxidase complex solution (ABC kit; Vector Laboratories, Burlingame, CA, USA) with 3,3-diaminobenzidine (Zymed Laboratories, San Francisco, CA, USA). The sections were then counterstained with Mayer's hematoxylin.

### RNA/protein extraction from paraffin embedded tissues

RNA/protein extraction was performed from formalin fixed paraffin-embedded (FFPE) muscular and femur tissues. RNA was extracted using an All prep DNA/RNA FFPE kit (QIAGEN, Hilden, Germany) for RT-PCR, and protein extraction was carried out using a Q proteome FFPE tissue kit (QIAGEN) following manufacturer's instructions for immunoblotting.

### Statistical analysis

Statistical analysis was performed using Prism 4.02 (GraphPad Software, San Diego, CA, USA). All data were evaluated using analysis of variance (ANOVA). All data were expressed as means ± standard deviations. The data were considered to be significantly different when p<0.05 or p<0.01 were determined.

## Results

### Effects of bioceramics on cell proliferation

**Figure 1:**
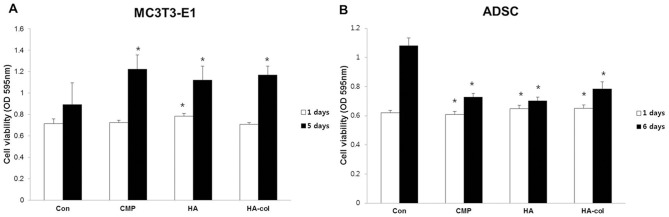
Effect of bioceramics on the proliferation of MC3T3-E1 cells (A) and ADSC (B). Bioceramics (CMP, HA and HA-col) were incubated for 24 h in standard medium (DMEM and α-MEM with 10% FBS and antibiotics) at 37°C, 5% CO_2_ in 6-well dishes. 2×10^2^ cells (ADSC and MC3T3-E1) were seeded in 96-well dishes, and the wells were filled with 100 μl medium. ^*^p<0.01 versus the control.

The effect of bioceramics on MC3T3-E1 cell and ADSC proliferation was assessed using the MTT-based viability assay. Direct exposure to bioceramics enhanced the proliferation rate of MC3T3-E1 cells and ADSC. MC3T3-E1 cells exhibited a marked increase in proliferation (40, 36, and 40% following incubation with CMP, HA, and HA-col, respectively) when compared to the control at 5 d. ADSC proliferation was increased by approximately 2-fold increase at 6 d compared to that in the control cultures. However, all the bioceramic-treated groups exhibited relatively enhanced proliferation (Figure 1).

### The ions released from bioceramics

Ion concentrations in the cell culture media changed as a result of bioceramics dissolution *in vitro* (addition of pen-strep and FBS). The PO_4_^3-^ ion concentration in the α-MEM + HA-col was increased compared to that in the α-MEM extract, although the difference did not reach significance (Table 2).

**Table 2:** Ion extract concentrations.

	Ca^2+^	PO_4_^3−^		Ca^2+^	PO_4_^3−^
α-MEM	68.36±0.67	37.92±0.67	DMEM	67.24±0.53	111.57±0.55
α-MEM + CMP	66.37±0.19	36.70±0.34	DMEM + CMP	67.37±0.43	33.06±0.56
α-MEM + HA	67.82±0.19	36.00±0.24	DMEM + HA	68.36±0.29	33.80±0.36
α-MEM + HA-col	62.34±0.19	99.45±0.13	DMEM + HA-col	69.41±0.28	34.49±0.58

(mg/kg = ppm).

DMEM and α-MEM ion concentrations were within 1% error.

The same lot of α-MEM and DMEM were used for all experiments.

### Gene expression in cells seeded in the bioceramics release medium

**Figure 2:**
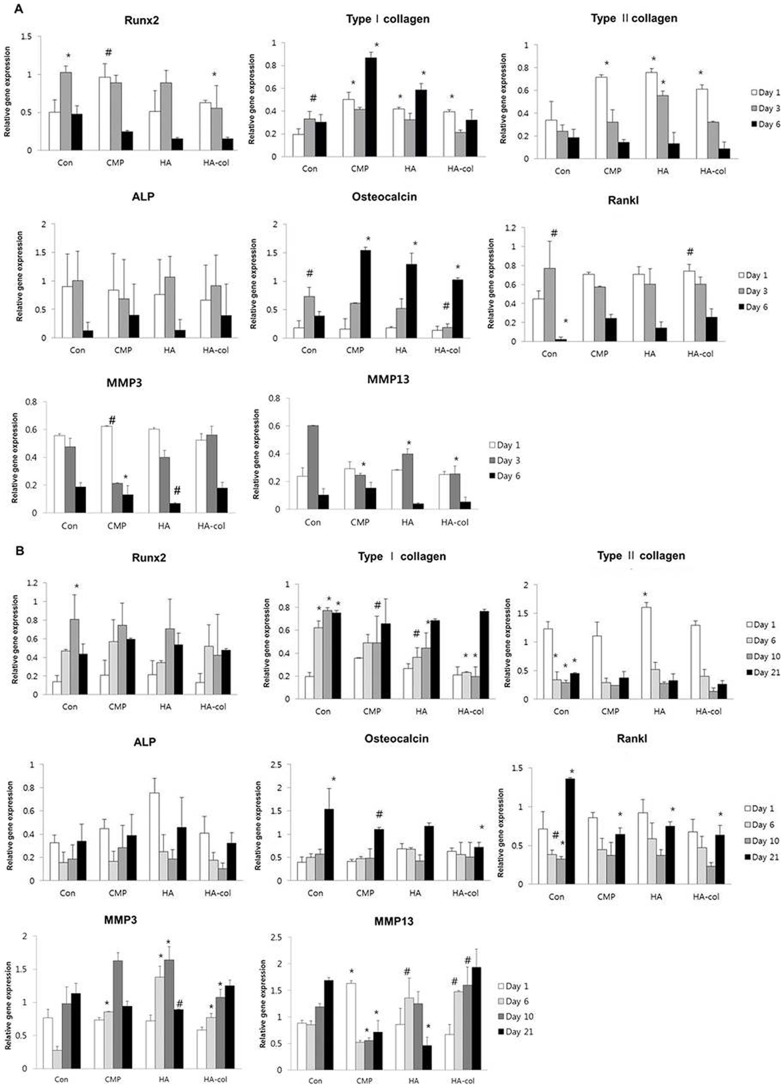
Quantitative analysis of the expression of genes involved in bone formation and bone resorption. mRNA expression of Runx2, type I collagen, type II collagen, ALP, osteocalcin, Rankl, MMP3, and MMP13 genes in MC3T3-E1 cells at 1, 3, and 6 d (A), and ADSC at 1, 6, 10, and 21 d (B) of culture in bioceramics release medium. Compared to the control, the expression level of type I collagen and osteocalcin of MC3T3-E1 cells was increased and those of Runx2, Rankl, MMP3 and MMP13 gene expression were decreased, each of which occurred in a time-dependent manner. Osteoblast dependent increases in osteogenic gene expression of Runx2, type I collagen, ALP and osteocalcin were observed for each bioceramic in ADSC cultures. Data are presented as mean ± standard deviation (n = 3). ^#^p<0.05, ^*^p<0.01.

The mRNA expression of genes related to osteogenesis after culture in either the bioceramics release medium or control medium is shown in Figure 2. The expression of osteogenic genes including type I collagen, ALP, Runx2, Rankl, osteocalcin, MMP3 and MMP13, was assayed by semi-quantitative RT-PCR. MC3T3-E1 cell cultures were examined at 1, 3, and 6 d while ADSC were examined at 1, 6, 10, and 21 d. Compared to control levels, there was an increase in the expression of type I collagen and osteocalcin, and a decrease in Runx2 and Rankl gene expression in MC3T3-E1 cells a time-dependent manner. Type I collagen and osteocalcin expression in MC3T3-E1 cells that were cultured on bioceramics increased significantly when compared to those in differentiation medium, as early as Day 1 of culture. This difference became more pronounced when the culture time was extended to 6 d.

Gene expression results for MC3T3-E1 cells are shown in Figure 2A. For Runx2, no significant difference was observed among cultures. Each of the bioceramic groups showed a relatively high level of type I collagen expression when compared to the control, particularly for the CMP samples, where expression increased by 4-fold at 7 d compared to that in the control. All groups exhibited a time-dependent decrease in type II collagen mRNA expression. Three bioceramic-cultured samples exhibited a high level of type II collagen expression at the initial stages that was about 3-fold higher than that of the control. No significant difference in ALP or Rankl mRNA levels was observed among any of the groups for the 6-day cultures. A significant increase in osteocalcin mRNA was observed in all the bioceramics samples at 6 d, however, MMP3 and MMP13 mRNA expression was decreased in all of the bioceramics samples at this time point.

Gene expression results for ADSC are shown in Figure 2B. Expression of Runx2 and type II collagen mRNA exhibited a similar pattern in all groups, including the control. The control group showed a moderately high level of type I collagen expression when compared to bioceramics groups for the entire experimental period, with the exception of Day 1. ALP and Rankl mRNA levels were highest at Day 1, decreased at the mid-time points, and rebounded at 21 d of culture. The expression of osteocalcin was markedly high at the 21-d time point in cells from all experimental groups. HA-col-treated cells exhibited an increase in MMP3 and MMP13 mRNA expression in a time-dependent manner. Conclusively, osteogenesis in all the three bioceramics samples was enhanced, corresponding to the osteogenic conditioned medium in the ADSC cultures.

### Immunoblot analysis of the bioceramics effects on the MC3T3-E1 cells and ADSC

**Figure 3:**
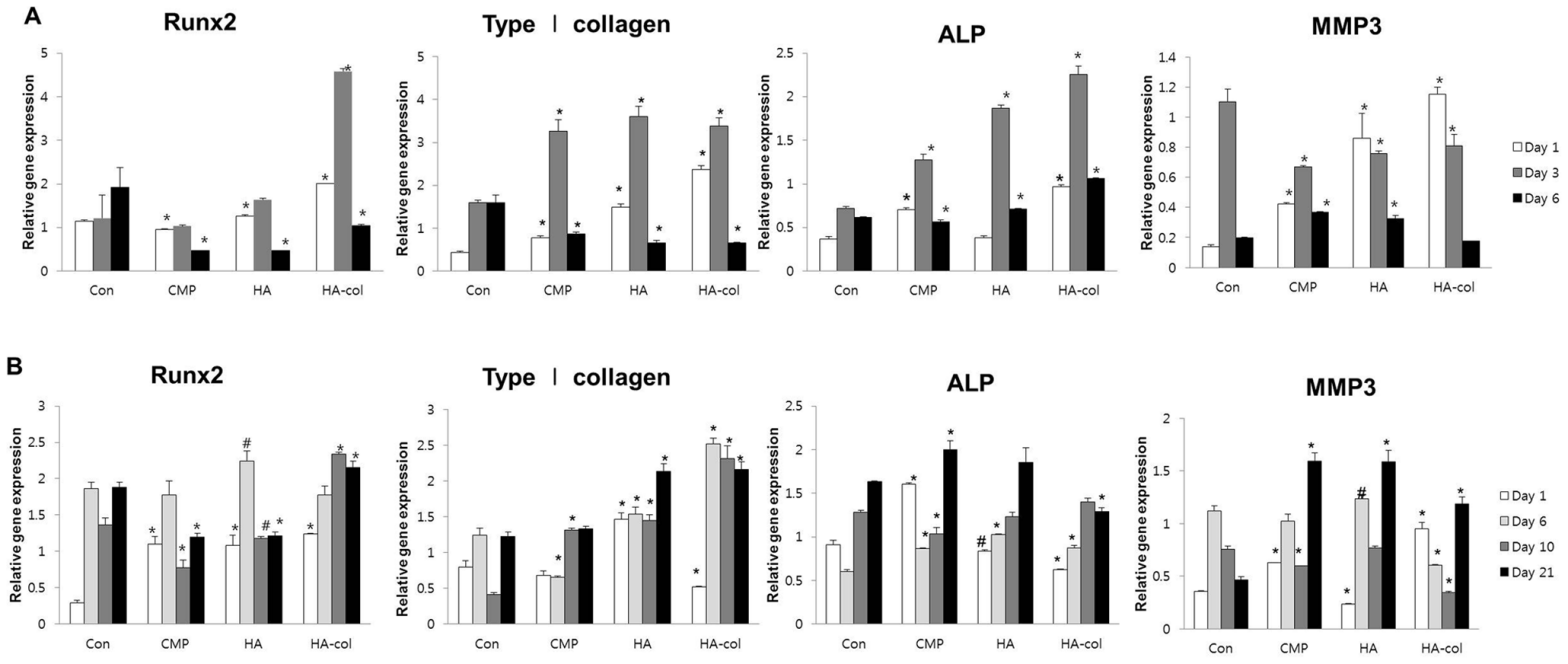
Immunoblotting for protein expression of osteogenic molecules in MC3T3-E1 cells (A) and ADSC (B). Relative ratios of Runx2, Type I collagen, ALP, MMP3 to β-actin were measured with Image J software. Data are representative of at least two experiments. Values are the mean ± standard deviation. Statistical difference as compared to corresponding control group. ^#^p<0.05, ^*^p<0.01.

Osteogenic gene-related protein expression after culture with bioceramics release medium and control medium are shown in Figure 3. The protein expression of osteogenic genes including type I collagen, ALP, Runx2 and MMP3 were assayed by immunoblotting MC3T3-E1 cells cultured for 1, 3, and 6 d (Figure 3A) and ADSC cultured for 1, 6, 10, and 21 d (Figure 3B) in each bioceramics release media. MC3T3-E1 cells showed a decrease in the expression of type I collagen, ALP, Runx2, and MMP3 at Day 6 when compared to the levels at Day 3. Runx2 and ALP were both similarly up-regulated in MC3T3-E1 cells cultured with bioceramics, with a peak increase at 3 d. The levels of Runx2 and type I collagen in ADSC cultured were high at 6 d, exhibiting a time-dependent increase. In contrast, the expression levels of Runx2, ALP and MMP3 in ADSCs increased at 21 d.

### Characterization of the bioceramics

**Figure 4:**
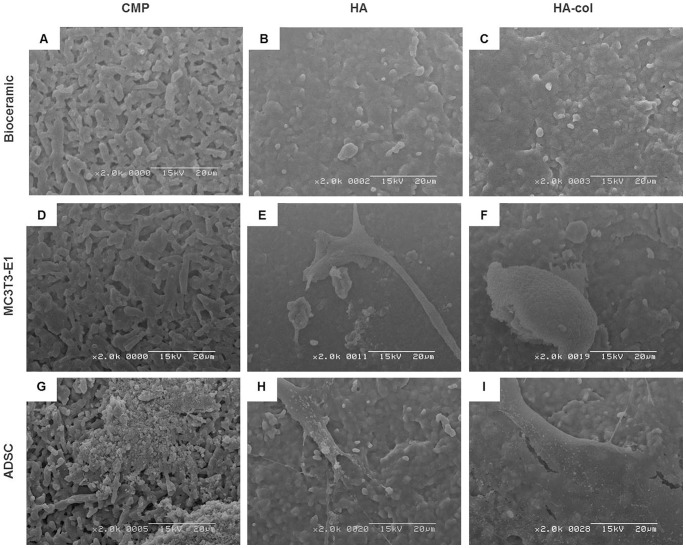
Scanning electron microscope observation of cell adhesion on bioceramic surfaces at 6 d of MC3T3-E1 cell culture, and at 10 d of ADSC culture. (A–C) Bioceramics only; (D–F) ADSC culture on bioceramic; (G–I) MC3T3-E1 culture on bioceramic. At 6 d of MC3T3-E1 culture, the cells were attached, exhibiting polygonal shapes on HA (E) and HA-col (F). At 10 d after ADSC seeding, the SEM images showed that cells were well attached on the surfaces of HA (H) and HA-col (I) with filopodia. Bioceramic: CMP, HA and HA-col. Scale Bar: 20 mm.

Bioceramic scaffolds were cultured with MC3T3-E1 cells or ADSC to assess morphology and adhesion on the bioceramics, and then examined by SEM at 6 and 10 d after seeding (Figure 4). SEM images of the surface morphology and microstructure of each bioceramic are presented in Figure 4A–C. At 6 d post-seeding, MC3T3-E1 cells were attached on the surface of the bioceramics, with the cells on HA and HA-col appearing much flatter and more spread out as compared to those on CMP (Figure 4D–F). In the case of the ADSC examined at the 10-days time point, cells grew well on all bioceramics (Figure 4G–I).

### Effects of bioceramics on the morphometric changes of MC3T3-E1 cells and ADSC

**Figure 5:**
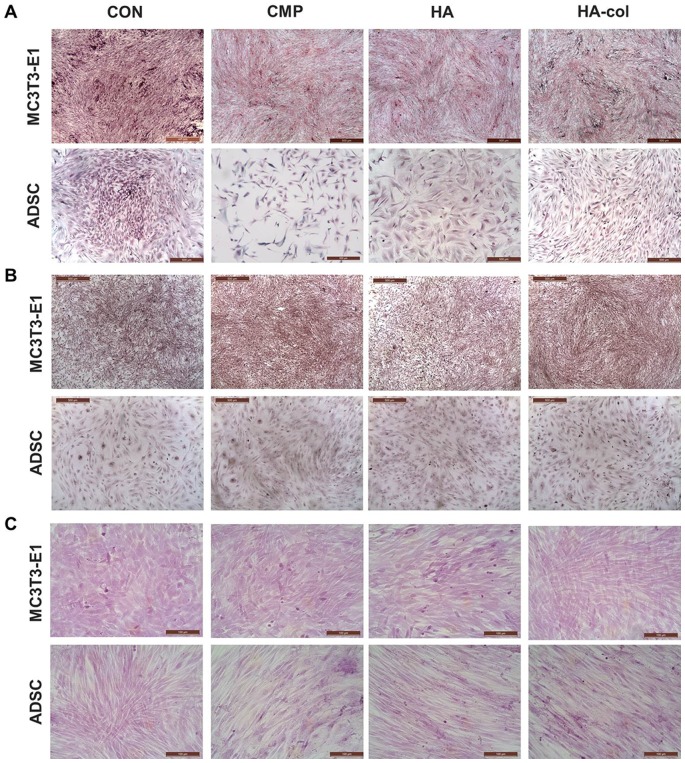
Cytochemical analysis shows extracellular matrix collagen and mineralization in ADSC cultures at 10 d, and MC3T3-E1 cell cultures at 6 d in bioceramic medium. Cells were plated in 6-well plates at a seeding density of 1×10^6^ cells/well, and were cultured for 6 and 10 d in a media containing ceramics. (A) Picrosirius staining shows the presence of type I collagen (red, orange, and yellow) in cell layers. (B) Alizarin Red S and (C) Von Kossa staining shows mineralization of the extracellular matrix.

It is well known that bioceramics induce the expression of type I collagen in MC3T3-E1 cells and ADSC [34], [35]. This enhanced collagen expression was observed extracellularly in the present study. Picrosirius staining of cell layers (Figure 5A) showed a greater density of type I collagen fibers in HA-col cultures when compared with those of CMP and HA for MC3T3-E1 cultures. In contrast, a higher density of fibrillar collagen was observed in CMP- and HA-released ADSC cultures when compared to HA-col cultures. This appearance is due to the birefringent nature of collagen fibrils in Picrosirius staining. Alizarin Red S (Figure 5B) and von Kossa (Figure 5C) staining were used to investigate mineralized matrix formation by bioceramics. The cytological results of MC3T3-E1 cells and ADSC cultures were negative in the present examination period of 6 and 10 d, respectively.

### Evaluation of radiopacity for newly formed tissues in bone defects

**Figure 6:**
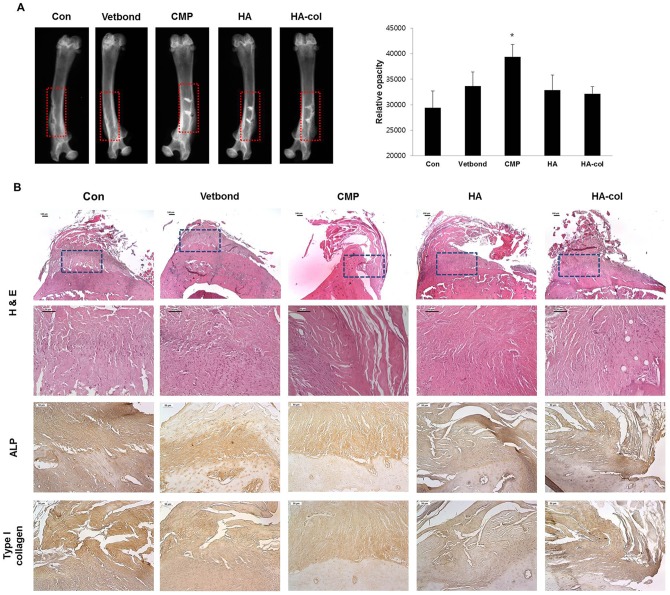
Results of radiopacity for the newly formed tissues after bioceramic implantation. Representative radiographs (A) and histological analysis (B) of a femur with bioceramics implantation for 12 weeks. (**A**) Density of the bone was analyzed by X-ray, and radiopacity levels were measured at the same area (red rectangle) from the bioceramics implanted region to the opposite side. The graph shows the relative opacity of X-ray films, which indicates the kind of implanted bioceramics. The stronger the radiopaque image in the red dashed rectangle, the higher the bone density. It is clear that the CMP sample exhibits higher radiopacity than the others. Vetbond, HA, and HA-col groups were at the same relative opacity, which was higher than that in the control. ^*^p<0.01 versus the control. (**B**) The cortical defect on the femur of all groups showed newly formed eosinophilic connective tissues. At higher magnification, the composition and density of the newly formed tissues of bioceramics were different from the control and Vetbond samples. Bioceramic-implanted groups revealed marked compact structures when compared to the others, which corresponds with the immunoreactivity of ALP and type I collagen. Representative H&E stained sections of bioceramic implants from 12 weeks after surgery. H&E, Magnification, ×100 and ×200. Immunohistochemistry for ALP and type I collagen, Mayer's hematoxylin counter staining, Magnification, ×200.

All groups, including the bioceramic-implanted groups and injury only groups, were analyzed by soft x-ray (radiographic analysis). All groups showed periosteal reaction at the injury sites with the control and Vetbond groups showing radiolucent areas at the injury sites. On the other hand, all bioceramic-implanted groups showed radiopaque lines near the implanted sites, with the CMP groups exhibiting the highest degree of bone regeneration. Further, the area between the femoral neck and implanted sites showed a trabecular pattern, which indicates that radiopaque materials infiltrated peri-implanted areas (Figure 6A).

### Histopathological and immunohistochemical findings in the implantation models

**Figure 7:**
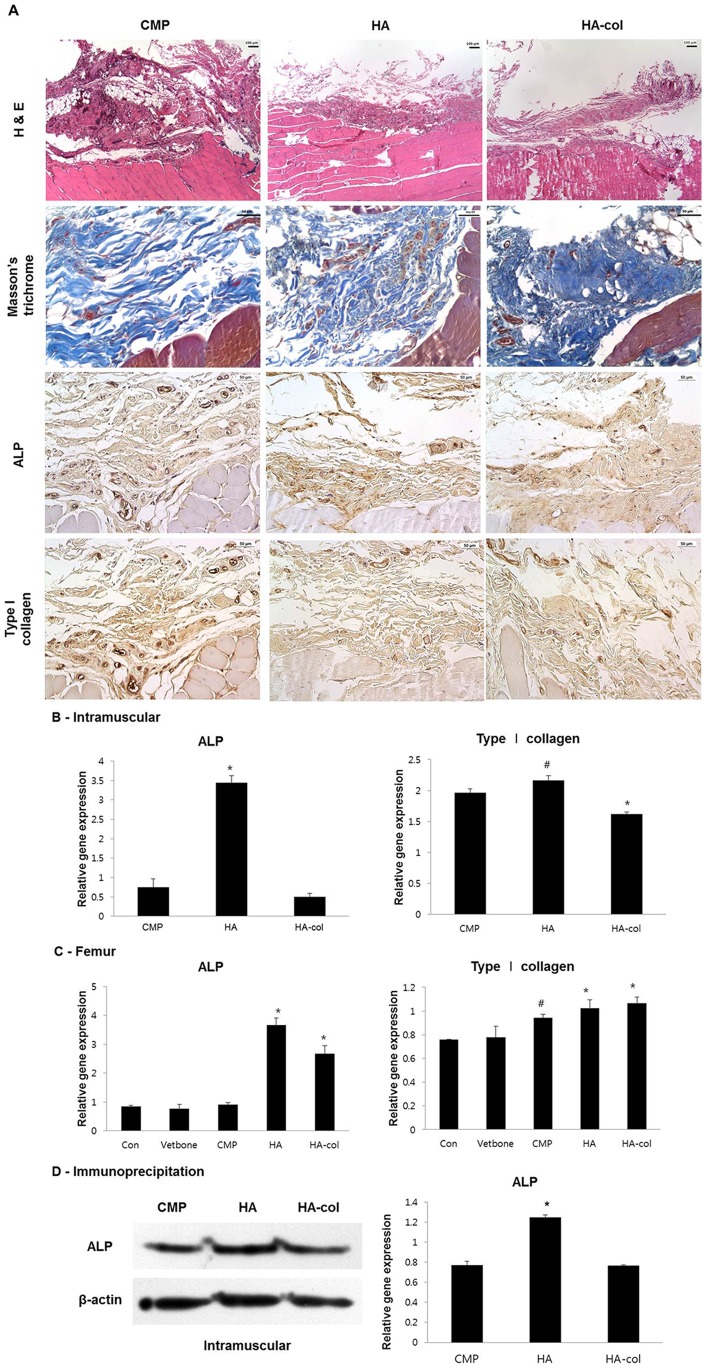
Histological analysis at 12 weeks post-implantation intramuscularly (A). In H&E staining, peripheral regions of the bioceramic-implanted muscles showed variable degrees of newly formed eosinophilic connective tissue deposition, which show a positive reaction to trichrome staining indicating a collagen-rich stroma. All bioceramic-implanted groups showed positive immunohistochemistry reaction to ALP and type I collagen but differences of positive intensity between each group were not obvious. Immunohistochemistry, Mayer's hematoxylin counter staining, Magnification, ×200. RT-PCR (B, C) for gene and immunoblotting for protein (D) expression of osteogenic molecules in the intramuscularis and cortical defects. (B, C) Formalin fixed paraffin-embedded (FFPE) muscular and femur tissue RNA was extracted by All prep DNA/RNA FFPE kit. In muscles, mRNA expression of ALP was increased to a greater degree in the HA group than in others, with similar results observed by immunoblotting as shown in Figure 6B. Type I collagen was up-regulated in the CMP and HA groups. (D) Expression of ALP was higher in the HA group than in the others, however, there was no significant difference in the expression level of type I collagen among all groups. Protein extraction from FFPE muscular tissues by Q proteome FFPE tissue kit for immunoblotting. Values are the mean ± standard deviation. ^#^p<0.05, ^*^p<0.01 versus the CMP group.

For all experimental periods, no significant change was observed in any of the animals. Fifteen rats were sacrificed at 12 weeks post-operatively. From histological analysis, newly formed eosinophilic connective tissues were detected in all of the bioceramics implanted intramuscularly with H&E staining (Figure 7A). Newly formed tissues were strongly positive for Masson's trichrome staining, indicating that the lesions were fully composed of collagen-rich stroma. The osteogenic capacity of newly formed collagen-rich stroma was evaluated by immunohistochemistry against ALP and type I collagen (Figure 7A), which were expressed at the early and late phases of osteogenesis, respectively. All bioceramic-implanted groups stained positive for ALP and type I collagen, but no obvious differences in staining intensity were observed among the groups.

The effects of bioceramics on the cortical bone defects were evaluated, and the region between each implanted area was observed by histology (Figure 6B). Newly formed eosinophilic connective tissues, similar to the intramuscular implants, were deposited at the lesion in the inter-implanted area. The composition and density of each group differed by degrees, especially in the bioceramic groups at higher magnification. In contrast to the variable density among all groups, immunoreactivity for ALP and type I collagen was not obvious like Figure 7A. These *in vivo* results corresponded to the osteoconduction and osteoinduction results *in vitro*, where minimal formation of mature bone with mineralization and lacunae was observed during the limited examination period of 12 weeks used in the present study.

### Gene/protein expression

RNA and protein from the newly formed collagen-rich tissues from muscle and bone implants were extracted using a FFPE tissue kit. For the muscular lesions, ALP mRNA was significantly increased among the groups, whereas type I collagen was not (Figure 7B). The protein level of ALP also exhibited a prominent increase in the HA (Figure 7D). ALP mRNA levels in HA-implanted femur lesions exhibited a 5-fold increase compared to the control and CMP levels (Figure 7C). The level of type I collagen in HA and HA-col was relatively increased (20 and 30%, respectively) when compared to that in other groups although all implantation groups were high level, including the Vetbond and bioceramic groups.

## Discussion

The ultimate objective of tissue engineering is to produce high-quality bone tissue *in vitro* that may be used as a clinical alternative to an autograft. Previous studies have been performed to investigate [36] the use of perfusion flow to increase the length of endothelial cell aggregations within co-cultures, osteoblast-specific gene expression, the cell number and coverage of the scaffold perimeter, and matrix area in the scaffold pores in the center of the scaffold [36]–[38]. Our current study demonstrated that adherence and proliferation of MC3T3-E1 cells and ADSC were not significantly different among the three evaluated bioceramics. MC3T3-E1 cells and ADSC cultured on bioceramics exhibited a marked expression of osteogenic mRNAs such as Runx2, type I collagen, and osteocalcin. Moreover, both ALP activity and calcium deposition were distinctly enhanced upon application of the bioceramics, as determined by the osteoblast activity [19], [39], [40]. Runx2 is able to induce both early and late markers for osteoblast differentiation, including ALP, type I collagen, osteopontin, bone sialoprotein, and osteocalcin in several cell lines. ALP and type I collagen were highly expressed in the early stage of bone maturation, whereas osteocalcin was expressed mostly at the later stages of osteogenesis [1], [9]. Genes involved in the bone resorption process, such as Rankl, osteoprotegerin G, and MMP13, are also regulated by Runx2 [41]–[43]. Osteoblast-derived MMPs function as a coupling factor and play important roles in bone turnover. MMPs are believed to generate collagen fragments, which are necessary for the recruitment and activation of osteoclasts and the initiation of bone resorption [44], [45]. The regulation of MMP13 is likely to have important consequences for both normal and pathological remodeling of bone where the balance between bone formation and bone resorption is disrupted [19], [46]–[48]. The calcium phosphate ceramics used in this experiment (i.e., CMP, HA, and HA-col) had a similar effect on the mRNA expression of type I collagen, Runx2 and osteocalcin in both MC3T3-E1 cells and ADSC. The increased osteocalcin expression was likely due to the increased expression of type I collagen at both the mRNA and the protein level. For MC3T3-E1 cells, all groups exhibited a distinct decrease of Runx2, MMP3, and MMP13 mRNA expression at the end of culture. ADSC cultured with bioceramics exhibited an increase in Runx2, MMP3, and MMP13 mRNA in a time-dependent manner. Bioceramics had no effect on the proliferation of MC3T3-E1 cells and ADSC by ion concentration release. MMPs exhibit favorable effects on cell adhesion, growth activities, and differentiation of osteoblasts. Runx2 has been found to be an essential transcription factor for inducing osteoblast differentiation and osteogenesis. Bioceramics were more effective in osteogenesis with increased collagen synthesis in the extracellular matrix. These results suggest that the solubility characteristics of bioceramics induced more suitable environments for differentiating osteoblasts, and thus provide useful information in our understanding of the biocompatibility and bioactivity of calcium phosphate ceramics, in spite of the lack of a distinct relationship to the concentrations of the released ions. Different cell types have been used in the present study to investigate osteoinductivity and osteoconductivity, such as those described in previous reports [38], [46], [48]–[50]. Our smart bioceramics, including CMP, HA, and HA-col with improved porous structures and mechanical properties, have successfully promoted osteoinduction and osteoconduction *in vivo,* as evaluated by intramuscular implantation and cortical defect femur models, respectively. There was no remarkable difference between each group with respect to histopathological findings. However, we identified that newly formed stroma-rich tissues from all bioceramic-implanted groups had positive immunoreaction for ALP and type I collagen, indicating that each of the bioceramics effectively promoted osteogenesis. In addition, results from RT-PCR and western blotting analysis using FFPE provided a more precise evaluation of differences among groups for osteogenic molecules. We observed that the HA group exhibited a greater increase of ALP mRNA than others. ALP is an early osteogenic marker, so ALP up-regulation indicates that HA could lead to osteoconduction in osteo-defects, and osteoinduction in extra-skeletal regions such as the muscles. The efficacy of bioceramics in promoting proliferation and differentiation toward bony tissues was similar among all groups. According to our results, culture with CMP resulted in faster rates of bone formation when compared to that with HA or HA-col. However, SEM results showed that cell adhesion rates were higher for HA and HA-col groups than for CMP groups, indicating that it is more efficient to use HA and HA-col than CMP bioceramics for cell therapy.

## Conclusions

This study shows that HA and HA-col bioceramics could be valuable biomaterials in bone regeneration studies by serving as an alternative to autografts and BMP therapies, suggesting an osteogenic potential for pre-clinical application. Our study provides useful information in understanding the biocompatibility and bioactivity of calcium phosphate ceramics.
